# Ethyl pyruvate reduces mortality in an endotoxin-induced severe acute lung injury mouse model

**DOI:** 10.1186/1465-9921-10-91

**Published:** 2009-10-02

**Authors:** Guan-Hong Shang, Dian-Jie Lin, Wei Xiao, Chong-Qi Jia, Yu Li, Ai-Hua Wang, Liang Dong

**Affiliations:** 1 Department of Respiratory Medicine, Qilu Hospital, School of Medicine, Shandong University, Jinan 250012, PR China; 2 Department of Respiratory Medicine, Shandong Province Hospital, School of medicine, Shandong University, Jinan 250012, PR China; 3 Department of Epidemiology and Health Statistics, Shandong University, Jinan 250012, PR China

## Abstract

**Background:**

Ethyl pyruvate (EP) was recently identified as an experimental therapeutic agent in a wide variety of model systems for inflammation-mediated tissue and cellular injury.

**Objective:**

To evaluate the effect of ethyl EP on improving the survival in mice with LPS-induced acute lung injury (ALI).

**Methods:**

ALI was induced by administering lipopolysaccharide (LPS) intratracheally. The mice were treated intraperitoneally (i.p.) with 100, 50 and 10 mg/kg EP immediately before intratracheal instillation of LPS, and 100 mg/kg EP was administered 0, 12, 24 and 48 hours after induction of ALI. The mortality rate was recorded and analyzed by the Kaplan-Meier method. Serum tumor necrosis factor (TNF)-α, interleukin (IL) -6 and IL-1 β were measured in bronchial alveolar lavage fluid using an enzyme-linked immunosorbent assay. High-mobility group box 1 levels were measured by Western immunoblotting.

**Results:**

Treatment with EP significantly inhibited the release of HMGB1, TNF-α, IL-6 and IL-1β into bronchoalveolar lavage (BAL) fluids of ALI mice, and reduced the permeability index of the injured lung. High EP doses reduced the mortality from ALI and the permeability index (100 mg/kg and 50 mg/kg EP versus control; P < 0.0001). Early administration of high-dose EP significantly increased survival rate (0, 12 and 24 h versus control; P < 0.0001, P < 0.0001 and P = 0.01 respectively by log-rank test). There was no survival advantage when EP was initiated at 48 h.

**Conclusion:**

Ethyl pyruvate improves survival and reduces the lung permeability index in mice with LPS-induced ALI.

## Introduction

Despite significant advances in understanding the pathogenesis of acute lung injury (ALI) and its management, the mortality rate from ALI remains unacceptably high [1,2]. It is well known that the pathogenesis of ALI is mediated by pro-inflammatory cytokines, including tumor necrosis factor (TNF)-α, interleukin (IL)-1β, IL-6 and high-mobility group box (HMGB) 1, which are released from macrophages, neutrophils and other cells of the innate immune system [3,4]. However, in two large clinical trails, administration of a monoclonal antibody against human TNF-α (TNF-α MAb) and a recombinant human IL-1 (rhIL-1ra) receptor antagonist failed to prolong survival in patients with sepsis syndrome [5,6].

HMGB1 is a late mediator of lethal systemic inflammation in animal models of cytokine-mediated disease. It is released by macrophages only after a delay of 12-18 h during endotoxemia [7]. Anti-HMGB1 antibodies protect against the lethal effects of LPS-induced endotoxemia in mice when administered 2 h after the onset of endotoxemia [7]. HMGB1 stimulates the release of TNF-α, IL-1β and other inflammatory cytokines from macrophages and pituicytes, and mediates ALI and lethality [8,9]. Moreover, in patients with severe infection, increased serum HMGB-1 levels correlated with non-survival [7]. These results suggest that HMGB1 is an important mediator in ALI and that its inhibition may be a key to improving clinical outcomes.

Ethyl pyruvate (EP) is a simple derivative of the endogenous metabolite pyruvic acid. Pyruvic acid is an end product of glycolysis, but it is also a potent antioxidant and free-radical scavenger. EP has been shown to improve survival and ameliorate organ dysfunction in a wide variety of animal models of severe sepsis [10], hemorrhagic shock [11], ischemia/reperfusion-induced intestinal mucosal injury [12], and ileus induced by bowel manipulation in mice [13]. EP inhibits lipopolysaccharide (LPS)-induced NF-κB activation in cultured RAW264.7 murine macrophage-like cells, and reduces HMGB1 release and TNF-α gene expression both in vitro and in vivo [10-13]. Therefore, we reasoned that EP might also be protective in established ALI, depending on inhibition of early and late inflammatory cytokines. The present study was designed to evaluate whether EP could be beneficial in a mouse model of LPS-induced ALI.

## Materials and methods

### Animals and Materials

Adult male BALB/c mice (22-25 g) were allowed to acclimatize for one week housed at 25°C on a 12-h light/12-h dark cycle; all animals were allowed free access to water and standard laboratory chow. All studies were conducted in accordance with the committee of Shandong University School of Medicine on the use and care of animals. The protocols were also approved by the Institutional Animal Care and Use Committee of Shandong University Qilu Hospital. All chemicals were purchased from Sigma-Aldrich (St. Louis, MO). Ethyl pyruvate solution was prepared as 28 mM EP, 130 mM NaCl, 4 mM KCl, 2.7 mM CaCl_2 _(pH 7.0).

### Animal Model of Lung injury

Before the induction of ALI, the mice were fasted overnight but allowed free access to water, then anesthetized with ketamine (100 mg/kg, i.m.) and xylazine (10 mg/kg, i.m.). To create the lung injury, 50 μg LPS from *Escherichia coli *(serotype O111:B4; Sigma-Aldrich) in 50 μl PBS was given intratracheally. Sham-operated mice underwent the same procedure with intratracheal injection of 50 μl PBS without LPS. To retrieve bronchoalveolar lavage (BAL) fluids, the airways were flushed with 1.0 ml PBS, BAL fluids were collected, and the permeability index as a quantitative marker of vascular leakage was determined as previously described [14]. In brief, bovine serum albumin (BSA) was labeled with ^125^I by the chloramine T method. A trace amount of ^125^I-BSA was added to unlabeled BSA (5 mg/ml in PBS), and 200 μl of this solution was injected intravenously. Four hours later, the mice were euthanized with ketamine and blood was collected from the inferior vena cava. The thorax was opened, the left atrium was incised, and the lung was perfused *in situ *with PBS via the pulmonary artery. The flushed lungs were removed and the permeability index (indicating the extent of pulmonary leakage) was determined using a gamma counter and expressed as the ratio of counts per min (cpm) in the whole lung to radioactivity in 100 μl of blood. BAL fluids were collected and the permeability index was calculated 12 h after ALI induction unless otherwise noted.

### Experimental Protocol

In the first experiment, 50 LPS-induced ALI mice were divided randomly into two groups (n = 25, each group). EP was administered intraperitoneally (i.p.) at 100 mg/kg immediately before intratracheal instillation of LPS, and control mice received the same dose of vehicle. HMGB1, TNF-α, IL-1β and IL-6 were measured in BAL fluids and the permeability indices were calculated.

The second experiment was designed to determine the effect on mouse survival of treatment with different doses of EP (100, 50 and 10 mg/kg) starting immediately before intratracheal LPS instillation. Four separate groups of mice (n = 30, each group) were used for this study. Among the 30 mice in each group, 20 were used to record mortality up to a week after the procedure and 10 were killed at 12 h to calculate the permeability index. The mice in these groups were treated with EP at 100, 50 and 10 mg/kg/d (i.p.), and the control mice received the same dose of vehicle until the end of the study. Mortality was recorded up to a week after the procedure (n = 20). Mice (n = 10) were killed and the permeability indices were calculated.

In the third experiment, the experimental and control mice were divided randomly into four treatment groups (n = 20, each group). EP was administered at the same dose (100 mg/kg/d, i.p.), and control mice received the same volume of vehicle beginning at four different time points (0, 12, 24 and 48 h after LPS instillation) until the end of study. Mortality was recorded up to a week after the injection.

### Cytokine Measurements

The concentrations of TNF-α, IL-6 and IL-1β were measured using commercially available enzyme-linked immunosorbent assay kits (R&D Systems, Minneapolis, MN) according to the manufacturer's instructions. The sensitivities for TNF-α, IL-6 and IL-1β were 15 pg/ml, 5 pg/ml and 5 pg/ml, respectively. Levels of HMGB1 were measured by Western immunoblotting as described by Wang et al. [7]. In brief, serum samples were ultrafiltered with Centricon 100 (Amicon, Millipore, Bedford, MA) to clear them of cell debris and macromolecular complexes, then concentrated 15-fold with Centricon YM-30 and separated on 12% sodium dodecyl sulfate-polyacrylamide gels. The separated proteins were transferred to an immunoblot polymembrane (Bio-Rad, Hercules, Calif), and HMGB1 was measured using polyclonal anti-HMGB1 antibodies (Santa Cruz, America, 1:500 dilution) and secondary anti-rabbit antibodies linked to horseradish peroxidase (Amersham Pharmacia, Buckinghamshire, UK). Standard curves were constructed using r-HMGB1 (Sigma-Aldrich Chemical), and the intensity of the 30-kd band was measured by densitometry.

### Statistical Analysis

Data are presented as mean ± SEM unless otherwise indicated. Differences between treatment groups were determined by Student's t test or 1-way analysis of variance (ANOVA) followed by the least-significant difference test, or Fisher's exact test. In the mortality study, time-to-survival data were analyzed by the Kaplan-Meier method and compared with the log-rank test; P < 0.05 was considered statistically significant.

## Results

The effect of ethyl pyruvate on pro-inflammatory cytokines in BAL fluids and permeability index of experimental ALI mice

To determine the levels of pro-inflammatory cytokines in BAL fluids, we flushed the airways with 1.0 ml PBS and collected the BAL fluids 12 h after ALI induction. As shown in Table 1, levels of the cytokines HMGB1, TNF-α, IL-6 and IL-1β were substantially elevated in BAL fluids from LPS-induced ALI mice (versus controls; P < 0.001).

**Table 1:** Cytokine concentrations in BAL fluids of ALI mice

Cytokine	Control	ALI	EP treatment	Unit
HMGB1	37.8 ± 10.8	129.0 ± 22.8*	86.4.8 ± 22.4^#^	ng/mL
TNF-α	377.4 ± 94.8	1584.3 ± 379.0*	650.5 ± 190.4^#^	pg/mL
IL-1β	86.0 ± 18.2	308.2 ± 50.5*	181.6 ± 53.5^#^	pg/mL
IL-6	82.9 ± 18.1	914.6 ± 103.5*	434.1 ± 126.0^#^	pg/mL

* P < 0.001 as compared with control group # P < 0.001 as compared with ALI group

**Figure 1:**
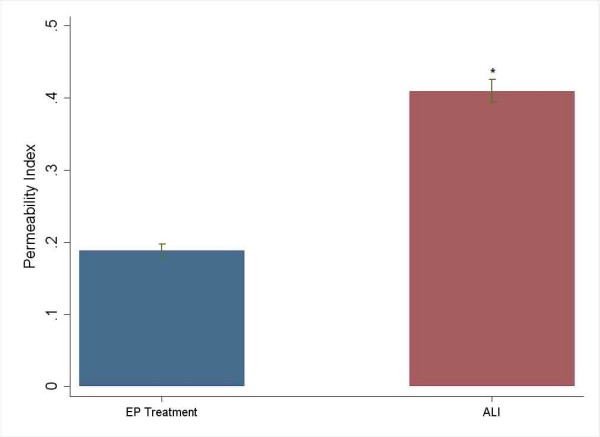
**The effect of ethyl pyruvate on permeability index in ALI**. *P < 0.0001 versus ethyl pyruvate treatment group.

Treatment with EP (100 mg/kg, i.p., immediately before intratracheal LPS instillation) significantly inhibited the release of HMGB1, TNF-α, IL-6 and IL-1β into the BAL fluids of ALI mice (Table 1), indicating that EP prevented LPS-induced ALI by attenuating the release of early (TNF-α, IL-6, and IL-1β) and late (HMGB1) systemic pro-inflammatory cytokines associated with lethality. As shown in Fig. 1, EP reduced the permeability indices of the injured lungs (Fig. 1).

Administration of high dose EP prevents the lethality of ALI and reduces the permeability index

**Figure 2:**
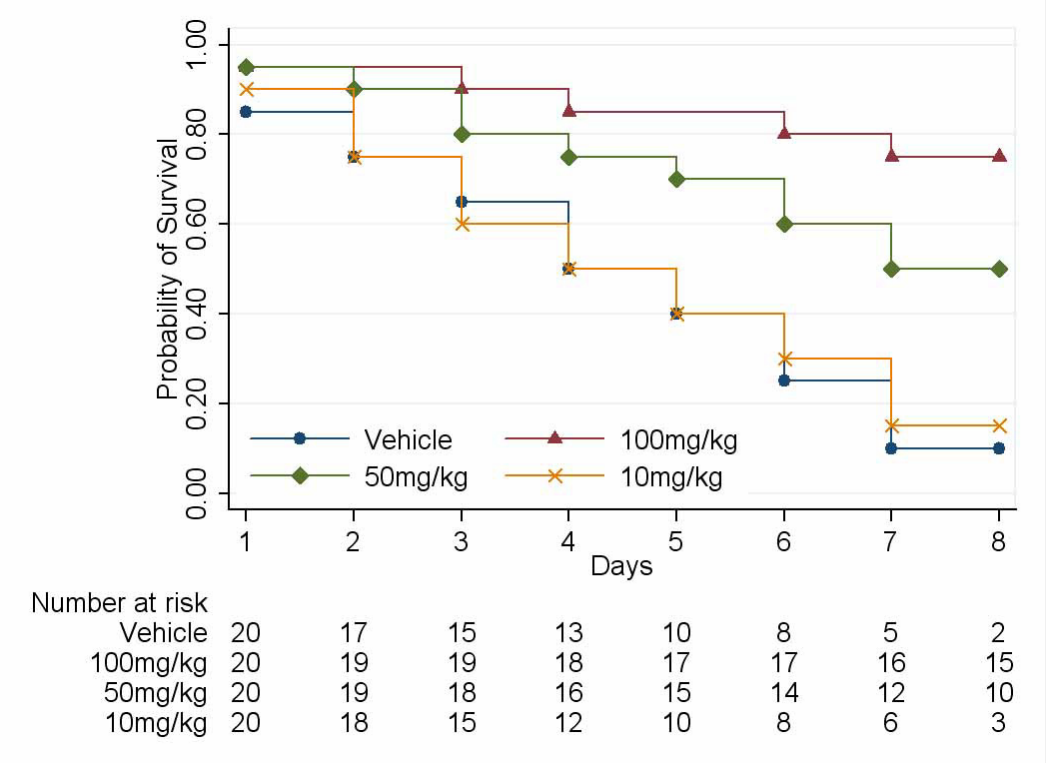
**Administration of high EP dose prevents the lethality of ALI**. Survival curves (100 mg/kg/d and 50 mg/kg/d EP versus survival in vehicle-treated controls; P < 0.0001 and P = 0.02 by log-rank test, respectively; 10 mg/kg/d EP versus vehicle-treated controls; P = 0.81 by log-rank test).

**Figure 3:**
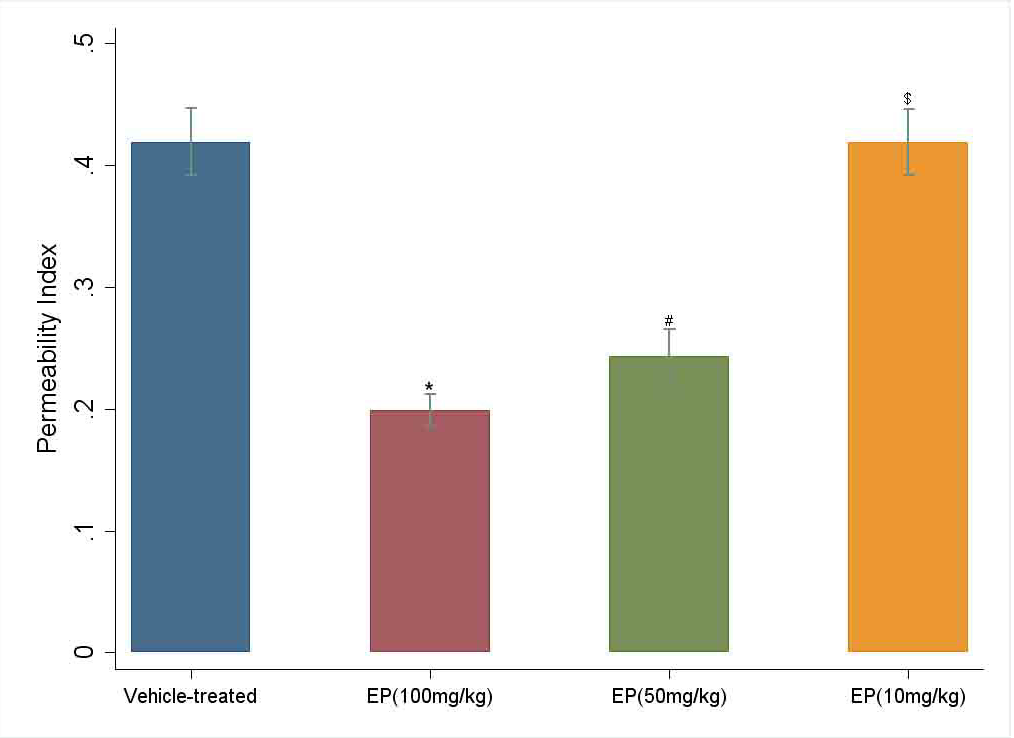
There was also a dose-dependent reduction in the permeability index (*^# ^P < 0.0001 versus vehicle-treated control; ^$ ^P = 1.00 versus vehicle-treated control).

To evaluate the protective effect of EP against lethality in LPS-induced ALI mice, the mice received three different doses of EP (100, 50 and 10 mg/kg/d. i.p.) starting immediately before intratracheal LPS instillation. The results showed that the high EP dose (100 mg/kg/d) protected against ALI lethality (survival in 100 mg/kg/d and 50 mg/kg/d of EP versus survival in vehicle-treated controls; P < 0.0001 and P = 0.02 respectively), but the effect was dose dependent; the low dose failed to protect significantly against death (survival in 10 mg/kg/d of EP versus survival in vehicle-treated controls; P = 0.81) (Fig. 2). Also, there was a dose-dependent reduction in the permeability index (100 mg/kg/d and 50 mg/kg/d EP versus vehicle-treated control, P < 0.0001; 10 mg/kg/d versus vehicle-treated control, P = 1.00; Fig. 3).

Early administration of high dose EP prevents ALI lethality

**Figure 4:**
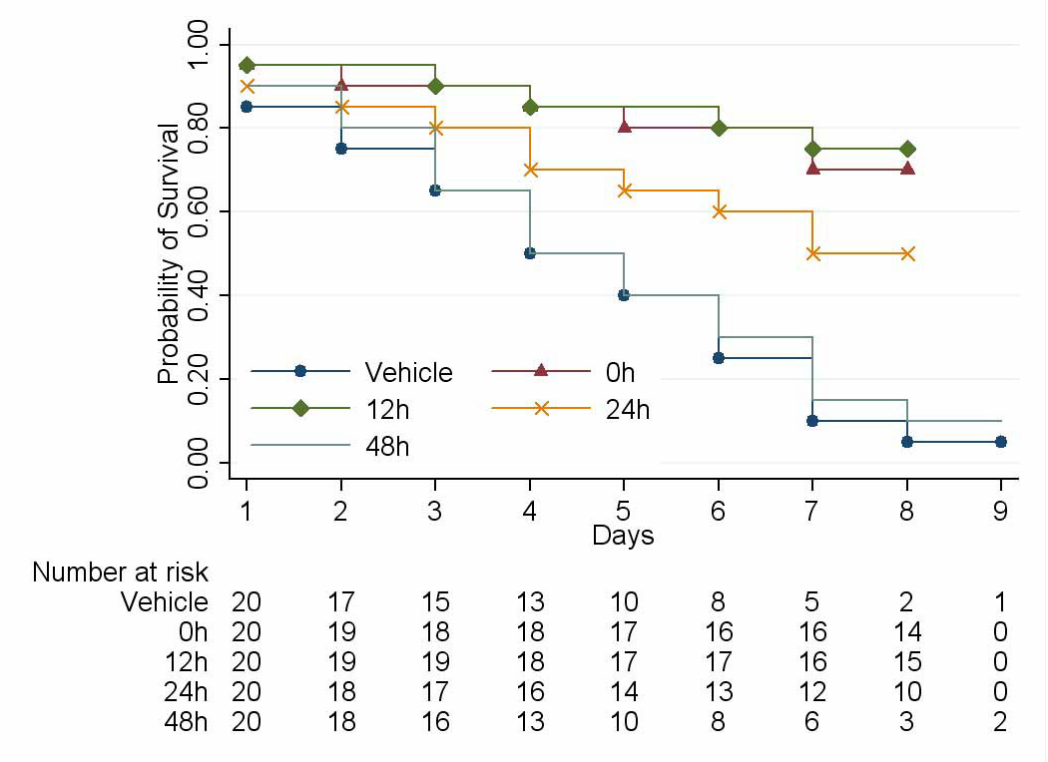
**Early administration of high dose EP prevents the lethality of ALI**. Survivals after administration of EP at 0, 12 and 24 h versus survival in vehicle-treated controls; P < 0.0001, P < 0.0001 and P = 0.01 by log-rank test, respectively; survival at 48 h versus survival in vehicle-treated controls; P = 0.75 by log-rank test).

To assess the therapeutic efficacy of early EP treatment against ALI, EP was administered at different time points (0, 12, 24 and 48 h after induction of ALI) at the same single dose of 100 mg/kg/d, i.p. The results showed that early administration of high dose EP (0, 12 or 24 h) significantly increased survival rate in the mice (survival after administration EP at 0, 12 and 24 h versus survival in vehicle-treated controls; P < 0.0001, P < 0.0001 and P = 0.01, respectively) (Fig. 4). However, no survival advantage occurred when EP was initiated at 48 h (survival after EP administration at 48 h versus survival in vehicle-treated controls; P = 0.75) (Fig. 4). Moreover, the animals treated with EP beginning at 0, 12, and 24 h were significantly more active and alert and fed more rapidly than either the animals with treatment beginning at 48 h or controls treated with vehicle.

## Discussion

Acute lung injury (ALI) is an important problem in humans and its pathogenesis is poorly understood. To investigate the molecular mechanisms of ALI, various experimental models have been used; intratracheal administration of LPS induces an ideal model because it results in lung injury without causing systemic inflammation and multi-organ failure. Moreover, the LPS-induced ALI model results in microvascular injury and diffuse alveolar damage with intrapulmonary hemorrhage, edema and fibrin deposition, which are also features of patients with ALI and acute respiratory distress syndrome (ARDS) [15,16].

In this present study, the levels of both early (TNF-α, IL-1β and IL-6) and late (HMGB1) cytokines in BAL fluids increased in mice with ALI 12 h after induction. Ethyl pyruvate (EP), an experimental pharmacological agent, significantly inhibited the systemic release of these cytokines, which mediate the lethality of ALI and systemic inflammation. Early administration of high-dose EP (0, 12, 24 h) significantly protected against ALI lethality. However, low dose EP or delayed administration gave no advantage in terms of survival. It is likely that pro-inflammatory cytokines, notably TNF-α, IL-1β and IL-6, participate in the early development of inflammation; they have been shown to play a crucial role in ALI and ARDS [17]. Persistent elevation of pro-inflammatory cytokines in serum and BAL fluid in ALI or sepsis patients is associated with a worse outcome [18]. Furthermore, instillation of IL-1β and TNF-α into the lungs leads to neutrophil accumulation, interstitial edema and histological changes consistent with inflammatory lung injury [17-19]. High concentrations of TNF-α are found in BAL fluids from patients with sustained ALI and ARDS, and high levels of IL-6 have been described in a number of acute conditions such as burns, major surgery and sepsis [20]. In patients with ALI and ARDS, non-survivors have significantly higher BAL-fluid-to-plasma cytokine concentration ratios than survivors [21], indicating the critical role of these cytokines in mortality.

These results suggest that the levels of HMGB1 in BAL fluids were greater in LPS-induced ALI mice than control mice. Moreover, administration of EP reduced the late mediator HMGB1. Unlike other pro-inflammatory cytokines, HMGB1 is a "late-appearing" inflammatory mediator, because its release during endotoxemia is delayed in comparison with the rapid increase of early pro-inflammatory cytokines such as IL-1β, IL-6 and TNF-α [7,10,22,23]. HMGB1 rose in the circulation starting at 8 h, increased until 16 h, and thereafter remained at a high level until 36 h during endotoxemia [7]. Further study showed that administration of anti-HMGB1 antibodies reduced lethality from 100% to 30% [7]. Intratracheal administration of HMGB1 produced acute inflammatory lung injury, and anti-HMGB1 Abs decreased the migration of neutrophils to the lungs as well as lung edema in endotoxin-induced acute lung inflammation [23]. Thus, delayed release of HMGB1 can participate in the downstream development of lung injury, and HMGB1 may be a distal mediator of acute inflammatory lung injury.

Ethyl pyruvate (EP), a very simple aliphatic ester derived from pyruvic acid, has been shown to be an effective anti-inflammatory agent in a wide variety of in vivo and in vitro model systems of inflammation-mediated cellular or tissue injury, including severe sepsis, hemorrhagic shock, ischemia/reperfusion-induced intestinal mucosal injury, and ileus induced by bowel manipulation in mice [10-13,24,25]. EP increases survival and reduces circulating levels of IL-6 [26]., and inhibits activation of NF-κB in Caco-2 human enterocyte-like cells stimulated with a mixture of TNF-α and IL-1β [27]. It blocks the secretion of HMGB1 by LPS-stimulated RAW 264.7 cells and the release of HMGB1 into the circulation in mice [10]. The survival advantage was apparent even when EP was administered 30 min before endotoxin infusion, and the treatment began 24 h after the onset of disease [10]. The molecular mechanism of EP action is to interfere with signal transduction through the p38 MAPK and NF-κB pathways, and to target directly the p65 subunit of the transcription factor [10,28]. EP inhibition of the p38 MAPK and NF-κB signal transduction pathways effectively induced the release of early (TNF-α, IL-1β and IL-6) and late (HMGB1) inflammatory mediators. Our data showed that administration of EP even when initiated 24 h after induction of ALI significantly increased survival, though no survival advantage occurred when administration was initiated at 48 h, when the levels of these cytokines had decreased significantly; indicating that EP modulation of HMGB1 and other inflammatory cytokines contributes to the beneficial effects on survival.

## Conclusion

In conclusion, EP decreased the release of pro-inflammatory cytokines and improved survival in mice with LPS-induced ALI. Thus, EP may be developed as a novel therapeutic adjunct in the treatment of ALI.

## Abbreviations

ALI: acute lung injury; EP: ethyl pyruvate; HMGB1: high-mobility group box 1; BAL: bronchoalveolar lavage; TNF: tumor necrosis factor; IL: interleukin.

## Competing interests

The authors declare that they have no competing interests.

## Authors' contributions

SGH designed the study, performed the analyses and wrote the manuscript. LDJ and XW helped to interpret the data and write the manuscript. JCQ helped with the statistics and writing. LY helped with the animal experiment and writing. WAH helped with the cytokine measurements, animal experiments and writing. DL performed experimental measurements and helped to write the manuscript.
